# Exhaustion of CAR T cells: potential causes and solutions

**DOI:** 10.1186/s12967-022-03442-3

**Published:** 2022-05-23

**Authors:** Taku Kouro, Hidetomo Himuro, Tetsuro Sasada

**Affiliations:** 1 Division of Cancer Immunotherapy, Kanagawa Cancer Center Research Institute, Yokohama, Japan; 2 Cancer Vaccine and Immunotherapy Center, Kanagawa Cancer Center, Yokohama, Japan

**Keywords:** Chimeric antigen receptor, Exhaustion, Tonic signal, Engineered cell, Epigenetics

## Abstract

Chimeric antigen receptor (CAR) T cell therapy has attracted attention for its promising therapeutic effects on hematological malignancies. However, there are problems such as relapse during long-term follow-up and limited effect on solid tumors with this therapy. Exhaustion, which impairs in vivo persistence and killing activity of CAR T cells, is one of the causes of these issues. Depending on their structure of extracellular portion, some CARs induce tonic signals in the absence of ligand stimulation and induce exhaustion phenotype in CAR T cells. Analysis of these self-activating CARs is expected to provide key information for understanding and resolving CAR T cell exhaustion. In this review, we introduced examples of self-activating CARs and summarized their phenotypes to figure out how CAR T cell exhaustion occurs. Further, we aimed to review promising solutions to the CAR T cell exhaustion that hampers generalized application of CAR T cell therapy.

## Introduction

**Fig. 1:**
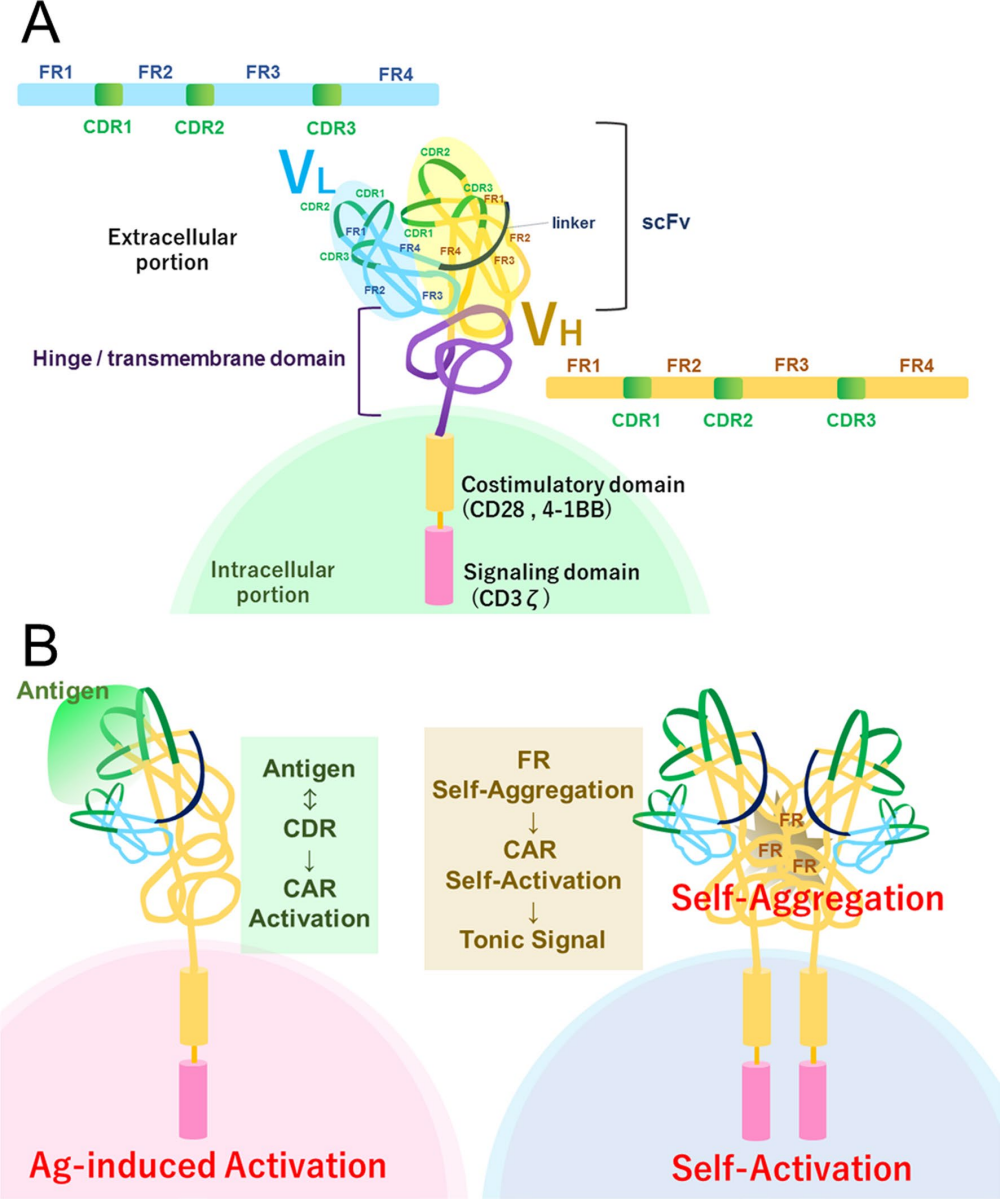
Schematic explanation of chimeric antigen receptor (CAR) structure and self-activation. **A** Basic structure of CAR. A CAR consists of three domains; extracellular, hinge/transmembrane, and intracellular domains. The extracellular domain of a CAR is the scFv containing a variable region of heavy chain (V_H_) and variable region of light chain (V_L_), which consist of four framework regions (FR1 ~ 4; blue/yellow) and three complementarity determining regions (CDR1 ~ 3; green). **B** Possible mechanism of CAR self-activation. CDRs are involved in antigen binding while FRs are associated with self-aggregation. By making hybrid of CDRs from the self-activating CAR of interest and FRs from non-self-aggregating CAR, self-activation can be avoided

The idea of combining the function of antibody with T cell receptor (TCR) arose from the fact that the affinity of the TCR for peptide-major histocompatibility complex (MHC) complex is lower than that of the antibody-antigen complex. Therefore, replacement of the TCR variable region with the variable fragment (Fv) of a monoclonal antibody is expected to induce a stronger signal in T cells. Indeed, such chimeric T cell receptors (cTCRs) successfully form TCR complexes, recognize their ligands, and induce IL-2 production and cell lysis in T cell hybridomas [1]. Furthermore, to avoid pairing of cTCR and endogenous TCR, a single chain format was introduced using the single-chain variable region fragment (scFv) of monoclonal antibody as well as a CD3ζ chain as a signaling domain [2], which is designated as a first-generation chimeric antigen receptor (CAR). As this type of chimeric receptor does not require endogenous CD3 molecules (γ, δ, ε, and ζ) in the signaling complex, CAR can be applied to T cells as well as NK cells. However, the first-generation CAR cannot induce the activation of naïve T cells [3] because they require a second signal from costimulatory molecules for activation. Therefore, the CD28 intracellular domain was introduced into the CAR construct to provide both the first and second signals from a single chain (28z-CAR) [4]. This is designated as the second-generation CAR and has been widely utilized in clinical trials and therapeutics (Fig. 1A). Even the second-generation CAR with CD28 co-stimulatory domain, however, cannot mimic TCR signal in effector/memory T cells, which utilize TNF receptor superfamily members such as OX40 and 4-1BB as costimulatory molecule [5]. This has led to the development of the second-generation CAR with 4-1BB costimulatory domain (BBz-CAR), and third-generation CAR, which possesses multiple costimulatory domains. Furthermore, fourth-generation CAR has been designed to modify tumor microenvironment (TME) by secreting cytokines such as IL-12 [6] and IL-15 [7, 8].

Albeit CAR is designed to mimic TCR signaling, there are a number of differences in the activation mechanisms between CAR and TCR. First, 28z-CAR does not induce significant phosphorylation of CD3 components other than ζ chain, because CAR is designed to work independently of endogenous TCR components. Furthermore, 28z-CAR phosphorylates LAT to a lesser extent than TCR [9]. Although CD3ζ is a major component of the CD3 complex, its phosphorylation alone is not sufficient for full activation of the ZAP-70–LAT pathway. In addition, in another second-generation CAR containing the 4-BB costimulatory domain (BBz-CAR), LAT phosphorylation was also not detectable [9]. The reason why CAR can function without phosphorylation of LAT, which is an essential scaffold molecule for TCR signaling, was explained by Dong et al. [10]. They used LAT-deficient cell lines expressing either TCR or CAR and found that only CAR, but not TCR, can form a ligand-induced microcluster. Thus, CAR might have a bypass pathway for activating downstream signals, which has not been revealed yet.

CAR T cells against CD19 are one of the most successful adoptive transfer therapies, with a complete remission rate of 55% at 10.6 months in acute lymphoblastic leukemia (ALL) [11] and 29% at 40 months in chronic lymphocytic leukemia (CLL) [12]. However, effectiveness of CAR T cells in other cancer types, especially in solid tumors, remains to be improved. One of the major problems associated with unsuccessful CAR T cell treatment includes exhaustion of CAR T cells that results in impaired CAR T-cell expansion and persistence in vivo [13, 14]. In this review, we focus on CAR T cell exhaustion, which impair their functions, and discuss strategies to prevent it.

## Exhaustion of CAR T cells

T cell exhaustion is characterized by the expression of checkpoint molecules as well as activation markers and a defect in effector functions, and was first described in chronic lymphocytic choriomeningitis virus infection [15]. It is induced by excessive antigen signaling and is considered to be one of the negative feedback mechanisms of T cell activation [16]. While it would be indispensable to stop immune responses at certain stages to prevent adverse effects such as autoimmune diseases [17], it also prevents the effects of cancer immunotherapy; tumor infiltrating T cells frequently become exhausted by excessive antigen stimulation and/or inhibitory TME [18]. Similarly, exhaustion is a common issue in CAR T cells that devastates their functions. For example, it was reported that anti-mesothelin CAR T cells in an orthotopic mouse model of pleural mesothelioma showed an exhausted phenotype represented by PD-1 upregulation and the defective expression of effector molecules such as IL-2, IFN-γ, TNF-α, and GRZB, resulting in impaired functions [19]. In addition, CD19 CAR T cells infused in patients with CLL [20] or B-cell lymphomas [21] tended to show exhaustion phenotype in non-responding patients. Furthermore, single cell RNA sequencing of CD19 CAR T infusion product also showed enrichment of exhaustion signature in patients with partial response or progressive disease compared to those with complete response [22]. These observations suggest that CAR T cell exhaustion is one of the major obstacles to successful treatment.

## Induction of CAR T cell exhaustion by tonic signaling

It has been recognized that nonactivated T and B cells at basal state transmit a low-level constitutive signal, designated as a tonic TCR or B cell receptor (BCR) signal, in the absence of ligands. Tonic signaling from TCR or BCR (including pre-BCR) in lymphocytes are involved in cell differentiation and maintenance of cellular responses to antigen stimulation [23, 24]. Similarly, in the case of CAR, various levels of ligand-independent receptor signaling caused by self-aggregation of CAR, also designated as tonic signal, have been observed [25]. Interestingly, higher levels of tonic signaling have been reported to result in the exhaustion and dysfunction of CAR T cells (Fig. 1B). The structure of CAR extracellular domain is thought to be responsible for tonic signaling. For example, CAR with IgG1 CH2-CH3 region as a spacer between the transmembrane domain and scFv generated a stronger tonic signal compared to that with CH3 only [26].

Some CAR T cells, such as c-Met IgG4 28z CAR T cells, show long-term in vitro proliferation without exogenous antigen stimulation or growth factors, which is designated as “continuous CAR”. In T cells expressing this “continuous CAR”, proliferation is considered to be mediated by self-activating tonic signal originating from CAR; for example, the c-Met IgG4 28z CAR in NFAT Reporter Jurkat cell line showed ligand-independent activation of the NFAT signal [27]. In addition, reduction of c-Met IgG4 28z CAR expression on cell surfaces ameliorated continuous proliferation, suggesting that ligand-independent activation of CAR signal is mediated by self-aggregation. Of note, these CAR T cells might be in the exhausted state in vitro because they show upregulation of T-bet and EOMES, which is associated with T cell exhaustion [28, 29]. Indeed, although c-Met IgG4 28z CAR T cells showed good cytotoxicity in vitro, their antitumor activity was compromised in vivo [27].

Another type of self-activating CAR T cells (GD2.28z) showed impaired proliferation and an exhausted phenotype in vitro [30]. This phenotype was attributed to the tonic CAR signaling activated by ligand-independent aggregation of CAR scFv, which was dependent on the framework region (FR) rather than on the complementarity-determining region (CDR) (Fig. 1A). Although GD2.28z CAR T cells showed strong cytotoxicity against target cells in vitro, their in vivo activity was very poor. In addition, GD2.28z CAR T cells upregulated the expression of checkpoint molecules such as PD-1, TIM-3, and LAG-3, which indicates exhaustion [31].

Interestingly, these two types of self-activating CARs induced different phenotypes in in vitro T cell proliferation. As mentioned above, c-Met IgG4 28z CAR T cells show continuous proliferation in vitro, but fail to persist in vivo, while GD2.28z CAR T cells exhibited poor proliferation both in vitro and in vivo. The differential in vitro proliferation capacity exhibited by the two functionally defective CARs may be explained by the difference in cytokine expression, because c-Met IgG4 28z CAR induced sustained IL-2 levels whereas GD2.28z CAR downregulated IL-2 expression. CAR signaling can upregulate *IL2* transcription through the activation of NFAT and NF-κB, but this process is suppressed by TIM-3 [32]. It is possible that differences in the strength of constitutive CAR signaling may lead to differences in the strength and/or duration of the exhaustion phenotype, including TIM-3 expression, which affects the expression of the *IL2* gene (Table 1).

**Table 1:** Comparison of chimeric antigen receptors (CARs) with and without tonic signaling

Antigen	CD19	c-Met	GD2
CAR construct	Second-generation with CD28	Second-generation with CD28	Second-generation with CD28
Ex vivo proliferation	~ 2 weeks without stimulation	Continuous without stimulation	Poor
Ex vivo killing activity	Good	Good	Good
IL-2 expression	Moderate	Increased	Defective
Ex vivo exhaustion	No	Upregulation of T-bet and EOMES	Upregulation of inhibitory molecules such as PD-1, LAG3 and TIM-3
In vivo persistence	Good	Poor	Poor
Reference	[33]	[27]	[9]

The characteristics of three types of CARs are compared. c-Met and GD2 CARs show tonic signaling, while CD19 CAR does not. Note that although c-Met and GD2 CARs show opposite phenotypes in some properties, they both indicate exhaustion to various extents

As the self-activating CAR T cells spontaneously fall into exhaustion, they might be a convenient and desirable model to examine the molecular mechanisms of CAR T exhaustion. For example, RNA sequencing (RNAseq) analysis of highly exhausted CAR T-cells expressing a high-affinity version of GD2.28z (HA.28z) showed markedly different gene expression patterns compared to those in CD19.28z CAR T cells. Upregulated genes in HA.28z CAR T cells included activation-related genes (*IFNG, GZMB,* and *IL2RA*), inhibitory receptors (*LAG* and *CTLA4*), and inflammatory factors (*CXCL8, IL13,* and *IL1A*), while downregulated genes included memory-related genes (*LEF1, TCF7, IL7R,* and *KLF2*) [34]. This gene expression profile overlapped with that observed in exhausted T cells following a chronic viral infection [35], suggesting the feasibility of this model for T cell exhaustion.

It has been speculated that the difference in gene expression between exhausted and non-exhausted CAR T cells may reflect differential chromatin accessibility. For example, an assay for transposase-accessible chromatin using sequencing (ATAC-seq) analysis of HA.28z CAR T cells showed enrichment of AP-1-bZIP and bZIP-IRF binding motifs, in accordance with upregulated expression of JunB, IRF4, and BATF3, which antagonize the function of classical AP-1 [34]. In addition, when a time course of chromatin accessibility status of exhausted and non-exhausted CAR T cells during ex vivo expansion was examined using the same model, differential chromatin accessibility was detected earlier than when differential expression of exhaustion markers was observed, suggesting a causal role of chromatin remodeling for the CAR T cell exhaustion phenotype [36]. Tumor-specific exhausted T cells were reported to show two distinct chromatin states: a plastic dysfunctional state from which T cells can be rescued, and a fixed dysfunctional state in which the cells are resistant to reprogramming [37]. Similarly, CAR T-cells with tonic signaling show two-stage exhaustion-related chromatin remodeling [38], and the exhaustion-imprinted epigenome in CAR T cells can be reversed by termination of the tonic signal [38] as discussed below.

## Strategies to avoid CAR T cell exhaustion induced by tonic signaling

### Modification of the structure of CAR extracellular domain

Self-aggregation of CAR that induces tonic signaling is mediated by the scFv portion of CAR. Thus, modification of scFv is a possible strategy to ameliorate tonic signaling by self-aggregation. As self-aggregation depends on the FR rather than CDR [30], CDR grafting into FR of non-aggregating scFv may be a good strategy to improve CAR. For example, this method has been experimentally utilized to exhausting GD2.28z CAR by constructing hybrid scFv composed of GD2 CAR CDRs and CD19 CAR FRs [30]. Although this approach involves creating a hybrid construct of two scFv regions, selection of a suitable FR is important but difficult. Since for CDR grafting, FR most resembling the original monoclonal antibody needs to be selected [39], FR from non-exhaustive scFv such as CD19 scFv might not always be applicable to every type of self-aggregating scFv. Therefore, identifying or fine-tuning a suitable FR for each exhaustive CAR may be indispensable.

In addition, other portions of the CAR can be modified to reduce tonic signaling. For example, anti-GD2 CAR was modified by shortening the VH sequence and changing the hinge and transmembrane domain of human CD8α instead of the Ig-derived hinge and CD28 transmembrane domain, resulting in reduced self-dimerization as well as lower expression of an exhaustion marker PD-1 [40]. Furthermore, modification of the spacer between scFv and transmembrane domains has been shown to change the tonic signal. CAR carrying an immunoglobulin spacer with CH3 alone, instead of that with both CH3 and CH2, mitigated Ag-independent tonic signaling and restored cytolytic abilities without exhaustion [26].

### Selection of a costimulatory domain

It has been suggested that the costimulatory domains of CAR are critical for the phenotype of CAR T cells [41, 42]. For example, the 4-1BB intracellular domain was shown to modify the phenotype of exhausting GD2.28z CAR T cells. Incorporation of 4-1BB intracellular domain (designated as GD2.BBz CAR), instead of CD28 intracellular domain of GD2.28z CAR, reduced the exhaustion phenotype and enhanced cytokine production in vitro, as well as prolonged T cell persistence in vivo. Persistent activating signaling from the CD28 intracellular domain is suggested to induce exhaustion of GD2.28z CAR T cells, whereas the 4-1BB intracellular domain produces a weaker costimulatory signal [43], which may be sufficient for T cell activation in the presence of ligands, but insufficient as an exhaustion signal. Indeed, compared with GD2.BBz CAR T cells, GD2.28z CAR T cells showed increased expression of genes encoding inhibitory receptors and exhaustion-related molecules, whereas GD2.BBz CAR T cells exhibited higher expression of memory-related transcription factors [30]. Similarly, modification of c-Met IgG4 28z “continuous” CAR through the introduction of 4-1BB intracellular domain (designated as c-Met IgG4 BBz CAR) was also shown to abrogate constitutive signaling and ligand-independent continuous growth [27]. Furthermore, comparison between CD28-based and 4-1BB-based anti-BCMA CAR demonstrated that 4-1BB-based CAR T cells exhibited less exhausted phenotype [44]. Collectively, these results suggested that use of the 4-1BB costimulatory domain might be one of the feasible approaches to prevent exhaustion, because it has been verified in multiple self-activating CARs.

A notable property of BBz-CAR, but not of 28z-CAR, is the activation of TRAF-dependent non-canonical NF-κB signaling pathway. It has been reported that naïve and activated T cells activate the canonical NF-κB pathway by using CD28 for costimulation, whereas effector and memory T cells activate the non-canonical NF-κB pathway by using CD27, CD30 OX40, or 4-1BB for costimulation [5]. Thus, the signaling of 28z-CAR and BBz-CAR may mimic that of TCR and costimulatory molecules in T cells. For example, BBz-CAR reproduced a signal for memory T cell differentiation [45] that prevents the exhaustion of CAR T cells [46]. In addition, the metabolic status of 28z- and BBz-CAR T cells were also shown to be different in a CD19-CAR T cell model. In this model, BBz-CAR induced oxidative metabolism and led to a central memory T cell phenotype, whereas 28z-CAR induced glycolytic metabolism and produced an effector memory phenotype [47]. Such differences in the metabolic status of cells may be related to exhaustion in CAR T cells.

In some clinical studies, advantage of CD19-BBz over CD19-28z in the context of CAR T cell persistence in vivo was argued*,* supporting the importance of costimulatory domains in the phenotype of CAR T cells [42, 48, 49]. Nevertheless, caution needs to be exercised in interpreting these results because in these studies CD19-BBz CAR (CTL019) used the CD8-derived hinge/transmembrane domain while CD19-28z CAR used the CD28-derived one [41]. Since the CD8-derived hinge/transmembrane domain was reported to provide reduced self-dimerization than CD28-derived one as mentioned above [40], difference in the hinge/transmembrane domains might affect the phenotype of CAR T cells in these studies. In a preclinical research, direct comparison between CD28 and 4-1BB costimulatory domains in the CAR with the same hinge domain demonstrated advantage of CD19-BBz CAR in terms of cell survival and persistence over CD19-28z CAR [50]. In contrast, apart from in vivo persistence, CD19-28z CAR showed more rapid tumor elimination than CD19-BBz CAR at low dose in a mouse model, suggesting that the 4-1BB costimulatory domain is not always better in all respects [51]. In the case where abundancy in tumor antigen is limited and there is less risk of self-activation, CD28 co-stimulatory domain may be a better choice. Since there have not been enough studies that demonstrate clear superiority of either CD28-costimulated or 4-1BB-costimuated CARs for controlling tumors [52], further research remains be done.

As an excessive signal from CAR induces the exhaustion of CAR T cells, it would be another method of avoiding exhaustion to modify the intracellular domains of CAR. For example, disabling distal two of the three CD3ζ immunoreceptor tyrosine-based activation motifs (ITAM) by deletion or Y to F mutation resulted in balanced fates of effector and memory CAR T cells [53]. In addition, single amino acid substitution in CD28 YMNM motif to YMFM exhibited reduced T cell differentiation and exhaustion as well as increased skewing toward Th17 cells [54]. Furthermore, the mutation of CD28 in CD19-28z CAR, which leave only PYAP domain of three CD28 functional domains (YMNM, PRRP and PYAP), showed a reduction of exhaustion-related transcription factors and genes, resulting in a significant survival advantage [55].

### Transient termination of tonic signaling

One potential strategy to stop tonic CAR signaling is to suspend CAR expression when it is unnecessary. For example, an interesting system where a destabilizing domain (DD) was introduced into the CAR construct was reported [56]. In this system, CAR expression was halted via degradation, and the administration of a stabilizing reagent for DD prevents CAR degradation and restores CAR expression. With this system, the memory-like phenotype can be restored in exhausted CAR T-cells through CAR degradation, indicating that CAR-induced exhaustion is a reversible phenomenon [38]. This system may also be useful as a safety device to control CAR T cell-mediated adverse events wherein CAR expression can be terminated by stopping drug administration [56]. Interestingly, inhibition of the epigenetic modifier EZH2 attenuates the reversal of the exhausted phenotype, suggesting that the exhaustion-associated epigenome is remodeled via EZH2 during the termination of tonic CAR signaling [38].

Introduction of DD to CAR construct for amelioration of exhaustion is obviously labor-intensive and reported only in a single preclinical study [38]. Interestingly, the same authors reported that, similar to such modification of CAR construct, CAR T cells can be also rested using a clinically available kinase inhibitor dasatinib that potently and reversibly inhibits proximal CAR signaling kinases, resulting in the reversal of the exhausted CAR T cell phenotype [38]. Thus, dasatinib treatment during ex vivo expansion of CAR T cells might be another promising strategy to restore exhausted CAR T cells without modifying CAR construct, although further examples are needed to show its general applicability.

### Modification of downstream signaling

As described above, the differential gene expression in exhausted CAR T cells with tonic signaling can be explained by the defective chromatin access of some transcription factors [34]. For example, HA-28z CAR T cells with the exhausted phenotype show dysregulated expression of canonical AP-1 transcription factor. These cells had a reduced ratio of canonical c-Fos/c-Jun heterodimer, which drives *IL2* transcription against the non-canonical JUNB, IRF4, and BATF3, which antagonizes canonical AP-1 expression and function. It should be noted that CAR T cells engineered to overexpress c-Jun for recovery of canonical AP-1 function were shown to have enhanced expansion potential, increased functional capacity, diminished exhausted phenotype, and improved anti-tumor potency in vivo [34]. Note that dysregulated expression of canonical AP-1 transcription factor is reported only with HA-28z CAR T cells and has not been verified in other self-activating CAR T cells.

Some transcription factors have also been shown to be directly related to exhaustion phenotypes in CAR T cells. For example, the expression of NR4A family of transcription factors is positively correlated with PD-1 and TIM-3 expression [57], in addition to its roles in the development of regulatory T cells [58]. Moreover, its binding motifs are enriched in differentially accessible regions of exhausted T cells [59]. Interestingly, in comparison to wild-type CAR T cells, the transfer of *NR4A1, NR4A2*, and *NR4A3* triple knockout CAR T cells to tumor-bearing mice decreased the proportion of highly exhausted CAR T cells among the tumor-infiltrating lymphocytes and led to a better prognosis [57]. Of note, since NR4A family members have redundant functions in inducing exhaustion, triple knockout of NR4A genes is necessary for improving the CAR T cell function. For clinical application, disruption of NR4A genes in the (autologous) CAR T cells would be the barrier. Although CRISPR-engineered T cells were tested in a clinical trial [60], we may need to wait until “off-the-shelf” CAR T cells are available to fully take advantage of this method.

## Strategies to avoid CAR T cell exhaustion induced by other cellular or environmental factors

In addition to self-activation by tonic signaling, other cellular or environmental factors have been reported to induce exhaustion in CAR T cells. Some strategies for the prevention of exhaustion are presented and discussed below.

### Blocking inhibitory receptors and cytokines

Sustained overexpression of the inhibitory molecule PD-1 is observed in exhausted CAR T cells [30], similar to that observed in CD8 T cells exhausted following chronic viral infection [61]. The inhibitory signal from PD-1 is considered to contribute to exhaustion, since PD-1/PD-L1 blockade by monoclonal antibodies results in better viral control and T cell responses in a chronic viral infection model [61] although it is controversially argued that the effects of PD-1/PD-L1 blockade may be transient and cannot reverse exhaustion-related epigenetic imprint [59]. Similarly, in a CAR T cell exhaustion model, treatment with a PD-1 blocking antibody or a dominant-negative PD-1 construct in CAR T cells can restore their function to a certain extent [19]. In a clinical trial, administration of the PD-1 inhibitor pembrolizumab did not alter the expansion, persistence, and cytokine production of GD2 CAR T cells in neuroblastoma [62], while another recent trial showed longer survival by co-administration of anti-mesothelin CAR T cells and pembrolizumab in patients with mesothelioma [63]. These inconsistent results might be explained by the possibility that CAR T cell exhaustion is differently affected by PD-1 signaling depending on CAR products. However, at this moment there is no clear evidence to explain this discrepancy and further investigation would be required.

In addition to inhibitory receptor ligands, cytokines secreted within the TME might inhibit immune cell functions. For example, TGF-β secreted from prostate cancer cells [64] was reported to induce exhaustion in effector memory T cells [65]. To avoid the immunosuppressive effects of TGF-β, dominant-negative TGF-βRII was introduced into CAR T cells against the prostate-specific membrane antigen (PSMA) in aggressive human prostate cancer-induced mouse models. It caused increased proliferation, enhanced cytokine secretion, resistance to exhaustion, long-term persistence, and tumor eradication in vivo [66].

### Optimizing T cell subset and differentiation stage

In general, CAR T cells are generated by introducing the CAR gene into peripheral blood mononuclear cells (PBMCs) activated with anti-CD3 and IL-2 treatment. Since, in this procedure, a mixture of CD4^+^ and CD8^+^ T cells at various differentiation stages are used, it is expected that differences in T cell subsets could result in different phenotypes of CAR T cells. Comparison of the T cell subsets for CAR T cell production revealed that the less-differentiated T cell populations perform better [67]. For example, introduction of CD19 CAR into stem cell memory T cells (T_SCM_), the most primitive subset of memory T cells with self-renewing capacity [68], resulted in enhanced in vitro proliferation activity as well as prolonged in vivo persistence and better tumor control compared to conventional CAR T cells [69]. Since CAR T cell exhaustion occurs in terminally differentiated effector T cells, employment of T cells in earlier stages may be beneficial for preventing CAR T cell exhaustion.

Another method to control T cell homeostasis and survival includes treatment with cytokines such as IL-15 and IL-7 [70]. Thus, ectopic expression of these cytokines with CAR was adopted to inhibit exhaustion in CAR [7, 8]. For example, co-expression of IL-15 with GD2 CAR was reported to retain the central memory/stem cell-like phenotype in CAR T cells and show reduced PD-1 expression [40], suggesting that this method can be applied to ameliorate CAR T cell exhaustion. It is noteworthy that the introduction of *IL-15Rα* in the CAR construct showed an advantage over the introduction of other signaling domains [71], suggesting the importance of IL-15R signaling pathway in the prevention of exhaustion.

## Summary

Despite the development and success of the CD19 CAR T cell therapy against leukemias, this approach has not met similar success in other tumor types, especially solid tumors. This may be explained, at least in part, by the possibility that CD19 CARs, specifically consisting of scFv derived from FMC63 clone, is a rare exception that does not generate tonic signals by itself. Such a characteristic of CD19 CAR might be a great advantage over other CARs, which show variable levels of tonic signaling and lead to exhaustion, not only during the ex vivo expansion but also in tumor tissues in vivo [30]. Even with CD19 CAR, however, exhaustion following continuous encounter with CD19 molecules on tumor cells is an obstacle for achieving long-term remission [20]. Therefore, substantial efforts are required to understand the mechanisms underlying CAR T cell exhaustion and overcome it with technological innovation.

**Fig. 2:**
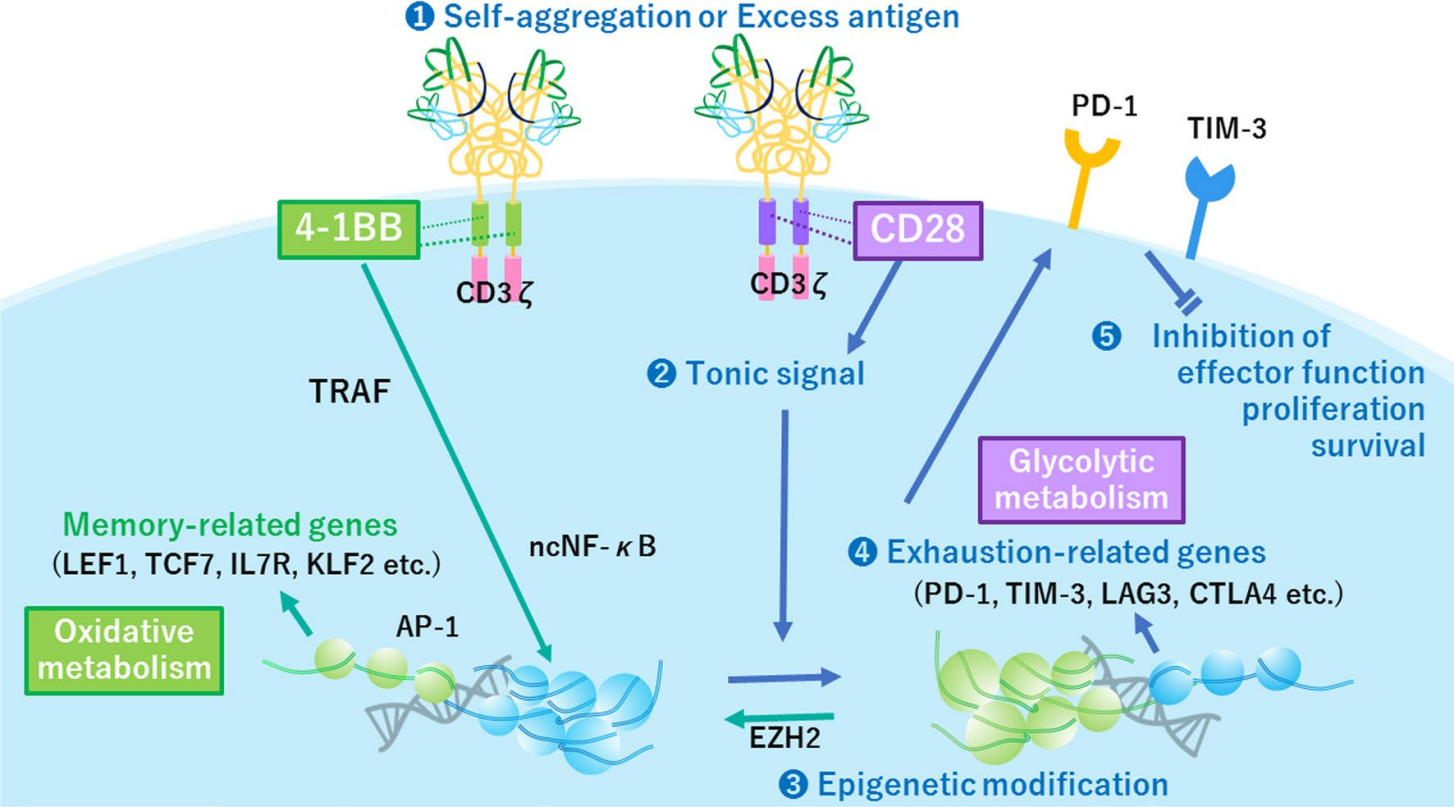
Signal transduction underlying chimeric antigen receptor (CAR)-induced exhaustion. Tonic signaling from aggregated 28z CAR induce the expression of exhaustion-related genes by modifying the epigenome. Inhibition of signals from inhibitory receptors does not revert the epigenomic status and recovers exhausted CAR T cells only transiently. The exhaustion-related epigenome can be reversed by the cessation of tonic signal emanating from CAR. BBz CAR generates TRAF-mediated non-canonical NF-κB (ncNF-κB) signal and induce memory T cell related gene expression

Recently, new technologies such as RNA sequencing and global chromatin landscape mapping by ATAC-seq have significantly contributed to the understanding of T-cell exhaustion. Reprogramming of the exhaustion-associated epigenome can be induced following cessation of CAR tonic signaling through several approaches [38] (Fig. 2). Selection of 4-1BB co-stimulatory domain might be a better approach for prevention of CAR T cell exhaustion when CAR has self-activating potency [27, 30, 44]. In addition, reduction of tonic signaling by modifying the scFv structure or transient termination of CAR expression can be considered as a promising approach to fine-tune CAR signaling. Thus, when scFv is modified, absence of tonic signaling should be included as one of the critical parameters when selecting suitable human framework region. In addition, identification of amino acid residues critical for tonic signaling may help fine-tune the framework structure. Furthermore, revealing the molecular mechanisms underlying exhaustion-related epigenetic remodeling and identification of molecular or pharmaceutical approaches to inhibit them will be a powerful measure for countering CAR T cell exhaustion.

At present, various mechanisms of CAR T cell exhaustion have been identified independently by different approaches. Although each study found responsible molecules, general mechanisms have not been identified. Therefore, there is no guarantee that one strategy effective for certain exhausted CAR is also effective for different one. Among various strategies to attenuate exhaustion of self-activated CAR T cells we have reviewed, which is the first choice when CAR T cells are found to be self-activated? First of all, use of the 4-1BB costimulatory domain might be promising way because this method has been verified in multiple self-activating CARs [27, 30, 44], although there has been no consensus for superiority of 4-1BB-derived domains over others yet. Alternatively, other methods, such as use of dasatinib in ex vivo expansion [38] and blockade of inhibitory receptors [63], could be attempted because these approaches are easier to perform without need to modify CAR constructs and has major merit especially when modification of CAR construct changes the stability of CAR protein, resulting in loss of its cell surface expression. Nevertheless, at this moment these approaches, including others, could not be actively recommended, because most of them were demonstrated in a single study, but have not been repeatedly verified in other studies. It would thus be future task to analyze various kinds of exhausted CARs in unified analysis protocol to clarify the differences and similarities among them. Such attempt will provide further improvement in effectiveness of CAR therapies.

## Data Availability

Not applicable.

## Abbreviations

CAR: Chimeric antigen receptor
TCR: T cell receptor
MHC: Major histocompatibility complex
Fv: Variable fragment
cTCRs: Chimeric T cell receptors
scFv: Single-chain variable region fragment
TME: Tumor microenvironment
ALL: Acute lymphoblastic leukemia
CLL: Chronic lymphocytic leukemia
BCR: B cell receptor
FR: Framework region
CDR: Complementarity-determining region
RNAseq: RNA sequencing
ATAC-seq: Assay for transposase-accessible chromatin using sequencing
ITAM: Immunoreceptor tyrosine-based activation motifs
DD: Destabilizing domain
PSMA: Prostate-specific membrane antigen
PBMC: Peripheral blood mononuclear cell
T_SCM_: Stem cell memory T cell
ncNF-κB: Non-canonical NF-κB
